# HIV drug resistance in persons who inject drugs enrolled in an HIV prevention trial in Indonesia, Ukraine, and Vietnam: HPTN 074

**DOI:** 10.1371/journal.pone.0223829

**Published:** 2019-10-10

**Authors:** Philip J. Palumbo, Yinfeng Zhang, Jessica M. Fogel, Xu Guo, William Clarke, Autumn Breaud, Paul Richardson, Estelle Piwowar-Manning, Stephen Hart, Erica L. Hamilton, Ngo T. K. Hoa, Mariya Liulchuk, Latifah Anandari, Tran Viet Ha, Kostyantyn Dumchev, Zubairi Djoerban, Irving Hoffman, Brett Hanscom, William C. Miller, Susan H. Eshleman

**Affiliations:** 1 Department of Pathology, Johns Hopkins University School of Medicine, Baltimore, MD, United States of America; 2 Vaccine and Infectious Disease Division, Fred Hutchinson Cancer Research Center, Seattle, WA, United States of America; 3 Frontier Science Foundation, Amherst, NY, United States of America; 4 Science Facilitation Department, Durham, NC, United States of America; 5 University of North Carolina Vietnam, Hanoi, Vietnam; 6 Gromashevsky Institute for Epidemiology and Infectious Diseases of the National Academy of Sciences of Ukraine, Kyiv, Ukraine; 7 University of Indonesia/Cipto Mangunkusumo Hospital, Jakarta, Indonesia; 8 Department of Health Behavior, Gillings School of Global Public Health, University of North Carolina at Chapel Hill, Chapel Hill, NC, United States of America; 9 Ukrainian Institute of Public Health Policy, Kyiv, Ukraine; 10 Departments of Hematology, Medical Oncology, and Medicine, University of Indonesia/Cipto Mangunkusumo Hospital, Jakarta, Indonesia; 11 Department of Medicine, University of North Carolina Chapel Hill School of Medicine, Chapel Hill, NC, United States of America; 12 Division of Epidemiology, College of Public Health, The Ohio State University, Columbus, OH, United States of America; International AIDS Vaccine Initiative, UNITED STATES

## Abstract

**Background:**

Persons who inject drugs (PWID) have high HIV incidence and prevalence, and may have limited access to antiretroviral therapy (ART) in some settings. We evaluated HIV drug resistance in PWID in a randomized clinical trial (HPTN 074). The study intervention included ART at any CD4 cell count with enhanced support for ART and substance use treatment.

**Methods:**

HPTN 074 enrolled HIV-infected PWID (index participants) with viral loads ≥1,000 copies/mL and their HIV-uninfected injection-network partners in Indonesia, Ukraine, and Vietnam; the study limited enrollment of people who reported being on ART. HIV drug resistance testing and antiretroviral (ARV) drug testing were performed using samples collected from index participants at study enrollment.

**Results:**

Fifty-four (12.0%) of 449 participants had HIV drug resistance; 29 (53.7%) of the 54 participants had multi-class resistance. Prevalence of resistance varied by study site and was associated with self-report of prior or current ART, detection of ARV drugs, and a history of incarceration. Resistance was detected in 10 (5.6%) of 177 newly diagnosed participants. Participants with resistance at enrollment were less likely to be virally suppressed after 52 weeks of follow-up, independent of study arm.

**Conclusions:**

In HPTN 074, many of the enrolled index participants had HIV drug resistance and more than half of those had multi-class resistance. Some newly-diagnosed participants had resistance, suggesting that they may have been infected with drug-resistant HIV strains. Behavioral and geographic factors were associated with baseline resistance. Baseline resistance was associated with reduced viral suppression during study follow-up. These findings indicate the need for enhanced HIV care in this high-risk population to achieve sustained viral suppression on ART.

## Introduction

Injection of illicit drugs carries a high risk of HIV transmission due to sharing of contaminated equipment. High HIV incidence and prevalence among persons who inject drugs (PWID) have been reported in many regions [1, 2]. Unfortunately, most PWID have limited access to HIV testing, antiretroviral therapy (ART), and other health services due to social stigmatization, criminalization of injection drug use, and other factors [3–5]. PWID often face barriers to HIV care, which can delay ART initiation [4]. ART has also been systematically withheld in some populations of PWID [6–8] due to concerns that PWID may be more likely to develop HIV drug resistance [9, 10] and that drug-resistant HIV will spread through drug-injection practices [10]. PWID often have unstable lives and may be less likely to adhere to ART, which would promote development of resistance [11, 12]. However, in some settings, PWID had similar rates of ART adherence and resistance to those who do not inject drugs [10, 13]. The frequency and patterns of resistance are important in this setting, since resistance impacts the ability of ART to suppress viral replication and prevent HIV transmission.

In this study, we analyzed baseline HIV drug resistance among PWID enrolled in a randomized, controlled, vanguard clinical trial in Indonesia, Ukraine, and Vietnam (HIV Prevention Trials Network [HPTN] 074) [14]. These study sites were selected because the HIV epidemics in these countries are concentrated among PWID [15, 16]; other factors considered in site selection were high HIV prevalence or incidence and the availability of medication-assisted treatment (MAT) for substance use. The goal of the HPTN 074 study was to assess the feasibility of performing future HIV prevention studies in PWID. The study enrolled active injection drug users, including HIV-infected index participants and their HIV-uninfected injection partners. Index participants were required to have HIV viral loads ≥1,000 copies/mL at study screening. The study limited enrollment of index participants who reported that they were on ART. Participants were randomized to a standard of care arm or an intervention arm. The intervention package was designed to increase use of local services for ART and MAT for substance use and included facilitated referral for ART at any CD4 cell count. Interventions for ART and MAT included systems navigation and counseling that encouraged engagement and retention in care and adherence to ART and substance use treatment. ART regimens were selected based on local treatment guidelines; recommended first-line ART regimens at the time of study included two nucleoside/nucleotide reverse transcriptase inhibitors [NRTIs] with a non-nucleoside reverse transcriptase inhibitor [NNRTI] [17]. After 52 weeks of follow-up, index participants in the intervention arm were more likely to report being on ART and MAT, were more likely to be virally suppressed, and had reduced mortality [14].

In this study, we evaluated HIV drug resistance and antiretroviral (ARV) drug use at study entry and the impact of baseline resistance on viral suppression. ARV drug use was assessed using self-reported data on ART and ARV drug testing. ARV drug testing data were used for the main study assessments, since we previously demonstrated that almost half (45.8%) of the index participants who had ARV drugs detected at study entry reported that they were not on ART, and 75% of those who reported that they were on ART did not have ARV drugs detected [18]. Findings from this study may help target resources to improve HIV care in these high-risk populations and inform the design of future research studies that include ART for HIV prevention.

## Methods

### Ethical considerations

The HPTN 074 trial protocol was approved by the following institutional review boards: Ukrainian Institute on Public Health Policy (Ukraine); Ethical Review Board for Biomedical Research Hanoi School of Public Health (Vietnam); Ethics Committee of Faculty of Medicine, University of Indonesia/Cipto Mangunkusumo Hospital (Indonesia); and the University of North Carolina Institutional Review Board. Written informed consent was obtained from all study participants.

### Study cohort

HPTN 074 (NCT02935296) enrolled HIV-infected index participants and their HIV-uninfected injection partners at three study sites (Jakarta, Indonesia; Kyiv, Ukraine; and Thai Nguyen, Vietnam; enrollment: 2015–2016). Enrollment criteria for index participants included: active injection drug user; HIV viral load ≥1,000 copies/mL at screening; and able to recruit at least one HIV-uninfected network injection partner; after the start of the study, index participants were also required to have a CD4 cell count >50 cells/mm^3^ at screening [14]. Exclusion criteria included participation in another HIV prevention study or HIV vaccine trial (previous or current), and appearance of a psychological condition, cognitive impairment, or other condition that would make participation unsafe. Study sites were instructed that people who reported that they were ART-naive should make up more than one half of the enrolled index participants. At enrollment, participants had face-to-face interviews with study staff. The interview questions included “Have you ever been on ART” and “Are you currently taking ART medications”.

### Laboratory testing

Baseline CD4 cell count testing was performed at study sites using local test methods. All other baseline laboratory results described in this report were obtained from retrospective testing performed at the HPTN Laboratory Center (Johns Hopkins University, Baltimore, MD, USA), including repeat HIV testing for quality control, viral load testing, HIV genotyping, ARV drug testing, and HIV-1 subtyping. Results from retrospective testing were not reported back to the participants. HIV testing was performed as described previously [14]. Viral load testing was performed using the RealTime HIV-1 Viral Load Assay (Abbott Molecular, Abbott Park, IL; lower limit of quantification: 40 copies/mL).

This study analyzed plasma samples from HIV-infected index participants collected at the enrollment visit; the analysis was limited to samples with viral loads >400 copies/mL to provide sufficient HIV RNA for analysis. HIV genotyping was performed using the ViroSeq HIV-1 Genotyping System v2.0 (Abbott Molecular, Des Plaines, IL). This system generates HIV *pol* gene sequences encoding HIV protease (amino acids 1–99) and reverse transcriptase (amino acids 1–335). Major HIV drug resistance mutations to NRTIs, NNRTIs, and protease inhibitors (PIs) were assessed using the ViroSeq resistance report. FASTA files were submitted to GenBank (accession numbers: MK228137-MK228585). HIV-1 subtyping was performed by phylogenetic analysis of ViroSeq-derived *pol* region sequences using PHYLIP v3.695 (http://evolution.genetics.washington.edu/phylip.html) and a set of 158 reference sequences including HIV-1 group M subtypes and recombinant strains from the database of the Los Alamos National Laboratory (https://www.hiv.lanl.gov). Results obtained using phylogenetic analysis were compared to results from online HIV subtyping tools: COMET v2.2 [19], REGA v3.0 [20], and RIP [21]. Agreement among at least three HIV subtyping methods was required to assign an HIV subtype. ARV drug testing was performed using a qualitative, high-throughput, assay that detects 20 ARV drugs in five classes (six NRTIs, three NNRTIs, nine PIs, one CCR5 receptor antagonist, and one integrase strand transfer inhibitor). The limit of quantification for the assay is 2 or 20 ng/mL, depending on the drug [22, 23]. Viral load testing was also performed in the main study using samples from the week 52 visit to assess viral suppression.

### Statistical methods

HIV-infected participants were classified as newly diagnosed with HIV infection if they did not report a prior positive HIV test result and had no ARV drugs detected. Exploratory analyses were conducted to identify potential associations between participant characteristics and two study outcomes: HIV drug resistance and viral suppression. Univariate logistic regression was used to estimate unadjusted associations (odds ratios) between participant characteristics and each of the two outcome variables of interest; multivariable logistic regression was used to estimate adjusted associations (adjusted odds ratios). Covariates were chosen *a priori* for the adjusted models based on their potential for confounding and/or effect mediation. Two covariates were chosen for the adjusted modeling of drug resistance: study site and ARV drug detection. Five covariates were chosen for the adjusted modeling of viral suppression: study site, ARV drug detection, baseline viral load, baseline drug resistance, and study arm. Geographic location (study site) is likely an important confounder due to its strong association with both outcome variables, and its correlation with many demographic and clinical characteristics. The remaining covariates are known to be associated with the respective outcome variables and therefore are expected to have relevant mediation effects. Statistical analyses were performed using SAS Version 9.4.

## Results

### Sample analyzed

**Fig 1:**
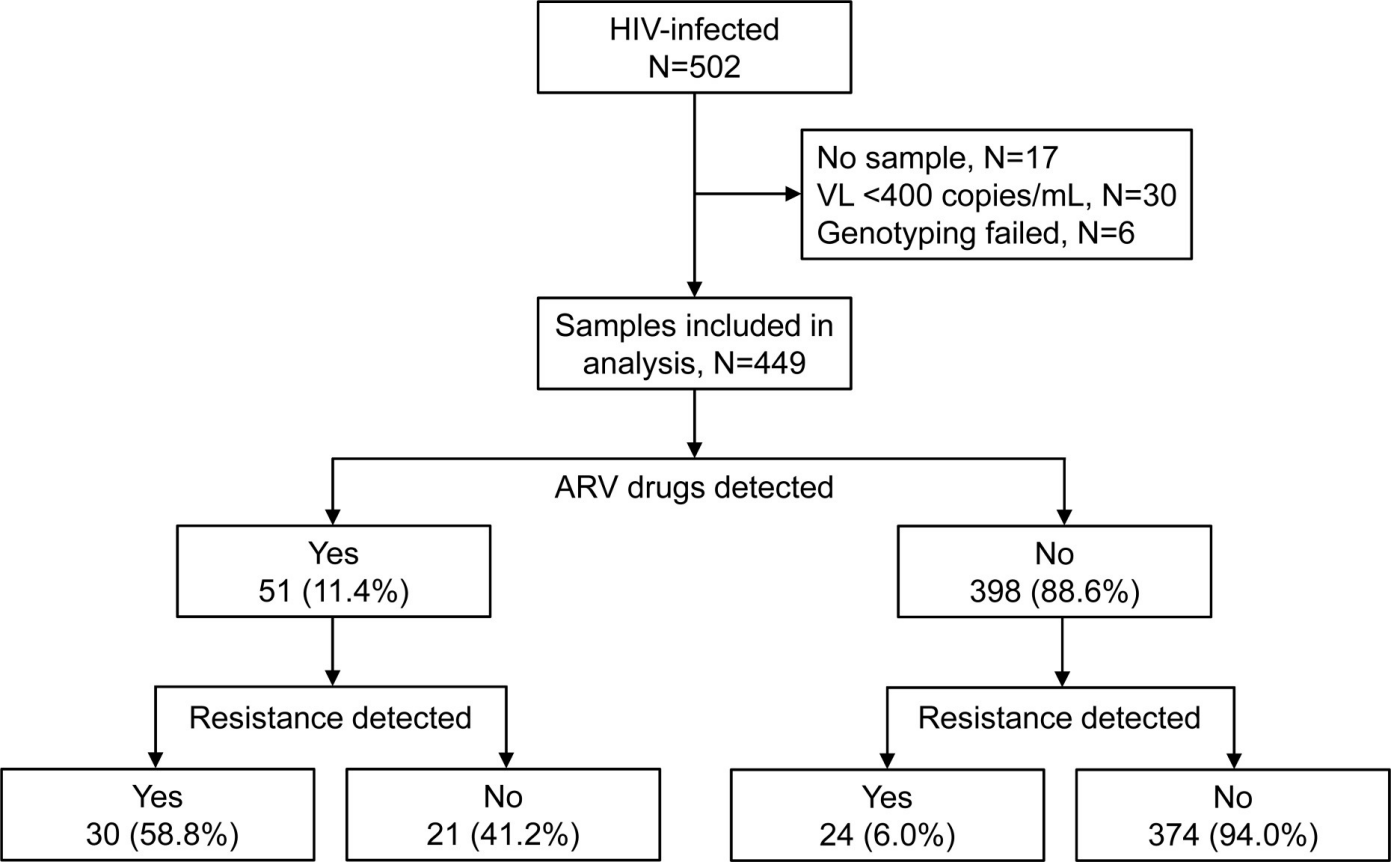
Overview of HIV drug resistance and antiretroviral drug testing. The figure shows a summary of the analysis of samples from HIV-infected participants enrolled in HIV Prevention Trials Network 074. HIV drug resistance detected refers to the detection of one or more major resistance mutations. Abbreviations: N, number; VL, viral load; mL, milliliter; ARV, antiretroviral.

Enrollment samples with viral loads >400 copies/mL were available from 455 (90.6%) of the 502 index participants in HPTN 074 (Fig 1). Results from HIV genotyping and ARV drug testing were obtained for 449 (98.7%) of those samples; 112 from Indonesia, 165 from Ukraine, and 172 from Vietnam. The HIV-1 subtypes identified were 60.6% CRF01_AE, 36.3% A1, 2.4% unique recombinant forms, and 0.7% B. The predominant HIV subtype in Indonesia and Vietnam was CRF01_AE (89.3% and 100.0%, respectively); the predominant subtype in Ukraine was A1 (98.8%). The prevalent HIV-1 subtypes detected were consistent with subtypes reported in Southeast Asia (CFR01_AE) and in Ukraine (A1) [24].

### HIV drug resistance

**Fig 2:**
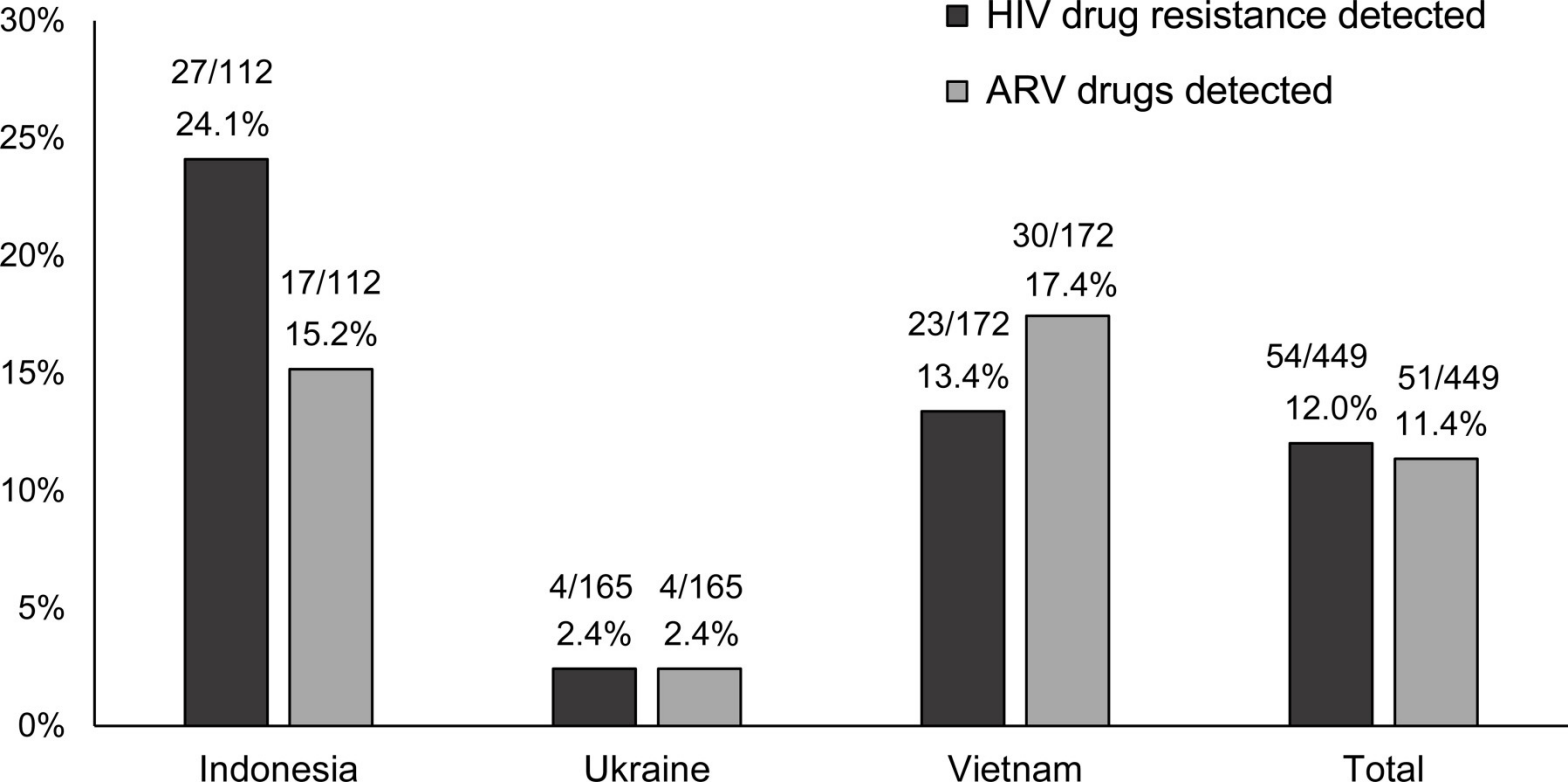
Prevalence of HIV drug resistance and antiretroviral drug use. The figure shows a summary of study findings. Results are presented by study site and overall (Total). The graph shows the proportion of participants with HIV drug resistance (dark bars) and the proportion of participants who had antiretroviral drugs detected (light bars). Abbreviation: ARV, antiretroviral.

Major HIV drug resistance mutations were detected in 54 (12.0%) of the 449 samples (27/112 [24.1%] in Indonesia; 4/165 [2.4%] in Ukraine; 23/172 [13.4%] in Vietnam; Fig 2). NNRTI resistance mutations were detected in all 54 cases (S1 Table). NRTI resistance mutations were also detected in samples from 29 (53.7%) of the 54 participants; 29 participants had multi-class resistance. No resistance mutations were detected that conferred resistance to PIs.

### Relationship between HIV drug resistance and ARV drug use

ARV drugs were detected in 51 (11.4%) of the 449 samples tested (17/112 [15.2%] from Indonesia; 4/165 [2.4%] from Ukraine; and 30/172 [17.4%] from Vietnam; Fig 2; Table 1). Drug resistance was significantly associated with detection of ARV drug, as noted below. Among the 51 participants who had ARV drugs detected, 30 (58.8%) had HIV drug resistance. In 30 cases, both ARV drugs and resistance were detected (Table 2; S1 Table, Group 1). The mutations K103N, Y181C, and G190A, which confer high-level resistance to NNRTI drugs efavirenz [EFV] and nevirapine [NVP], were detected alone and in combination in all 30 participants; EFV or NVP was detected in samples from 29 (97%) of the 30 participants. Twenty-four (80%) of the 30 participants also had resistance mutations to NRTIs (mostly M184V); 22 of the 24 participants also had NRTI drugs detected (mostly lamivudine [3TC]).

**Table 1:** Patterns of antiretroviral drugs detected.

	Indonesia (N = 17)	Ukraine (N = 4)	Vietnam (N = 30)	Total (N = 51)
**1 NNRTI**	**5**	**-**	**5**	**10**
EFV	2	-	4	6
NVP	3	-	1	4
**1 NNRTI + 1 NRTI**	**6**	**1**	**16**	**23**
EFV + 3TC	4	-	14	18
EFV + FTC	-	1	-	1
NVP + 3TC	2	-	2	4
**1 NNRTI + 2 NRTIs**	**5**	**2**	**7**	**14**
EFV + 3TC + TFV	3	-	7	10
EFV + 3TC + ZDV	-	1	-	1
EFV + 3TC + ABC	-	1	-	1
NVP + 3TC + ZDV	2	-	-	2
**2 NNRTIs + 1 NRTI**	**1**	**-**	**1**	**2**
EFV + NVP + 3TC	1	-	1	2
**Boosted PI + 1 NRTI**	**-**	**-**	**1**	**1**
LPV/RTV + 3TC	-	-	1	1
**Boosted PI + 2 NRTIs**	**-**	**1**	**-**	**1**
LPV/RTV + TFV + FTC	-	1	-	1

The table summarizes the findings from antiretroviral (ARV) drug testing. Results are shown by study site and overall (Total). The values indicate the number of participants with each pattern of ARV drugs detected. Abbreviations: N, number; NNRTI, non-nucleoside reverse transcriptase inhibitor; EFV, efavirenz; NVP, nevirapine; NRTI, nucleoside/nucleotide reverse transcriptase inhibitor; 3TC, lamivudine; FTC, emtricitabine; TFV, tenofovir; ZDV, zidovudine; ABC, abacavir; PI, protease inhibitor; LPV, lopinavir; RTV, ritonavir.

**Table 2:** Frequency of HIV drug resistance by antiretroviral drug use.

	Resistance detected			
	Overall	Indonesia	Ukraine	Vietnam
**Reported prior ART**	29/85 (34.1%)	18/32 (56.3%)	2/26 (7.7%)	9/27 (33.3%)
**Reported current ART**	21/42 (50.0%)	13/18 (72.2%)	0/1 (0%)	8/23 (34.8%)
**ARV drugs detected**	30/51 (58.8%)	14/17 (82.4%)	0/4 (0%)	16/30 (53.3%)
**Newly diagnosed**^**a**^	10/177 (5.6%)	6/45 (13.3%)	0/23 (0%)	4/109 (3.7%)

^a^ Participants were classified as newly diagnosed with HIV if they did not report a prior positive HIV test result and had no ARV drugs detected.

Twenty-three participants had ARV drugs detected, but did not have resistance mutations corresponding to one or more of the classes of drugs detected. One had PIs and 3TC detected, but only had NNRTI resistance; one had EFV and 3TC detected, but only had NNRTI resistance. The remaining 21 participants had ARV drugs detected (NNRTIs and/or NRTIs) with no resistance (S1 Table, Group 2). Because these participants were not virally suppressed, they were at risk of acquiring resistance to the additional classes of ARV drugs detected. Among the 398 participants with no ARV drugs detected, 24 (6.0%) had resistance (S1 Table, Group 3); nine (37.5%) reported a history of ART, including two who reported that they were on ART at study enrollment.

We next evaluated the relationship between self-reported ART and HIV drug resistance. Among 85 participants who reported prior ART and 42 participants who reported current ART, 29 (34.1%) and 21 (50.0%) had resistance detected, respectively (Table 2). Among 364 who did not report prior or current ART, 25 (6.9%) had resistance detected. Participants who reported that they had ever been on ART were more likely to have resistance than those who reported that they had never been on ART (29/85 [34.1%] vs. 25/364 [6.9%], p<0.001). Participants who reported that they were on ART at the enrollment visit were also more likely to have resistance than those who reported that they were not on ART at enrollment (21/42 [50.0%] vs. 33/407 [8.1%], p<0.001).

### HIV drug resistance among newly diagnosed participants

Among the 449 participants included in the study, 197 (43.9%) did not report a prior positive HIV test; 177 (89.8%) of the 197 participants had no ARVs detected. The 177 participants in this group were classified as newly diagnosed. HIV drug resistance was detected in samples from 10 (5.6%) of the 177 newly-diagnosed participants (Table 2).

### Factors associated with HIV drug resistance

We next evaluated the association between demographic, behavioral, clinical, and laboratory characteristics with HIV drug resistance (Table 3). The proportion of participants with resistance was significantly higher among those with ARV drugs detected compared to those with no ARV drugs detected in both univariate and multivariable models (p<0.001 in both models). The frequency of resistance also differed by study site (greater in Indonesia vs. Ukraine or Vietnam) and was higher among participants who reported a history of incarceration. Other factors were significantly associated with resistance in univariate models, but not in the multivariable models (CD4 cell count, injection amphetamine use, non-injection stimulant use, and hazardous alcohol use).

**Table 3:** Baseline factors associated with HIV drug resistance.

Variables	Number with resistance (%)	Unadjusted		Adjusted^a^	
		OR (95% CI)	P	aOR (95% CI)	P
**HIV viral load** (log_10_ copies/mL)^b^		0.71 (0.49, 1.03)	0.068		
**CD4 cell count** (cells/mm^3^)^c^		**0.73 (0.61, 0.87)**	**<0.001**		
**Antiretroviral drug(s) detected**					
No	24/398 (6.0%)	REF		REF	
Yes	30/51 (58.8%)	**22.26 (11.12, 44.55)**	**<0.001**	**22.31 (10.12, 49.19)**	**<0.001**
**Sex**					
Male	49/387 (12.7%)	REF			
Female	5/62 (8.1%)	0.61 (0.23, 1.58)	0.306		
**Age**					
18–29	12/77 (15.6%)	REF			
30–39	36/295 (12.2%)	0.75 (0.37, 1.53)	0.432		
40–60	6/77 (7.8%)	0.46 (0.16, 1.29)	0.139		
**Study site**					
Indonesia	27/112 (24.1%)	REF		REF	
Ukraine	4/165 (2.4%)	**0.08 (0.03, 0.23)**	**<0.001**	**0.08 (0.03, 0.24)**	**<0.001**
Vietnam	23/172 (13.4%)	**0.49 (0.26, 0.90)**	**0.022**	**0.50 (0.27, 0.94)**	**0.032**
**Marital status**					
Married/Have partner but not married	22/221 (10.0%)	REF			
Single/Divorced/Separated/Widowed	32/228 (14.0%)	1.48 (0.83, 2.63)	0.186		
**Education**^**d**^					
None or primary school	8/55 (14.5%)	REF			
Secondary school	25/225 (11.1%)	0.73 (0.31, 1.73)	0.480		
Higher education	21/169 (12.4%)	0.83 (0.35, 2.01)	0.684		
**Injected amphetamines**^**e**^ (3 months prior)					
No	53/390 (13.6%)	REF			
Yes	1/58 (1.7%)	**0.11 (0.02, 0.82)**	**0.031**		
**Non-injection stimulant use**^**e**^^,^^**f**^ (3 months prior)					
No	27/298 (9.1%)	REF			
Yes	27/150 (18.0%)	**2.20 (1.24, 3.91)**	**0.007**		
**Non-injection opiate use**^**e**^^,^^**g**^ (3 months prior)					
No	49/416 (11.8%)	REF			
Yes	5/32 (15.6%)	1.39 (0.51, 3.77)	0.521		
**Hazardous alcohol use**^**h**^					
No	45/298 (15.1%)	REF			
Yes	9/151 (6.0%)	**0.36 (0.17, 0.75)**	**0.007**		
**MAT for substance use**					
No	14/135 (10.4%)	REF			
Yes	40/314 (12.7%)	1.26 (0.66, 2.41)	0.480		
**Number of sexual partners** (1 month prior)					
0	29/183 (15.8%)	REF			
1	24/240 (10.0%)	0.59 (0.33, 1.05)	0.074		
≥2	1/26 (3.8%)	0.21 (0.03, 1.63)	0.136		
**Number of injection partners** (3 months prior)					
1	2/54 (3.7%)	REF			
2–4	44/340 (12.9%)	3.86 (0.91, 16.43)	0.067		
≥5	8/55 (14.5%)	4.43 (0.89, 21.90)	0.068		
**Jail/Prison** (ever)					
No	48/435 (11.0%)	REF		REF	
Yes	6/14 (42.9%)	**6.05 (2.01, 18.17)**	**0.001**	**3.59 (1.16, 11.12)**	**0.027**

The table shows factors associated with the detection of HIV drug resistance among index participants enrolled in HPTN 074. The analysis includes 449 index participants who had baseline results for resistance testing and antiretroviral drug testing. Logistic regression models were used to estimate adjusted associations between participant characteristics and resistance. P-values <0.05 are bolded. For the adjusted models, results are shown for variables that were significantly associated with HIV drug resistance after adjustment for other variables.

^a^ For each variable, univariate associations were adjusted in a multivariable model including covariates: study site and baseline ARV drugs detected (yes/no).

^b^ Assessed for increments of log_10_ HIV RNA copies/mL. The mean viral load among those with antiretroviral (ARV) drugs detected was 3.84 log_10_ copies/mL (standard deviation [SD]: 0.83); the mean viral load among those with no drugs detected was 4.30 log_10_ copies/mL (SD: 0.76). This difference was significant (p<0.001). The mean viral load among those with resistance was 4.08 log_10_ copies/mL (SD: 0.77); the mean viral load among those without resistance was 4.28 log_10_ copies/mL (SD: 0.78). This difference was not significant (p = 0.068).

^c^ Assessed for increments of 100 CD4-positive cells/mm^3^. The mean CD4 cell count among those with ARV drugs detected was 222 cells/mm^3^ (SD: 192); the CD4 cell count among those with no drugs detected was 344 cells/mm^3^ (SD: 210). This difference was significant (p<0.001). The mean CD4 cell count among those with resistance was 235 cells/mm^3^ (SD: 189); the CD4 cell count among those without resistance was 344 cells/mm^3^ (SD: 211). This difference was significant (p<0.001).

^d^ Secondary school includes completion of some or all of secondary school. Higher education indicates some or complete technical training or college/university education.

^e^ Data for substance use was missing for one participant.

^f^ Stimulants include cocaine and methamphetamines.

^g^ Opiates include heroin and opium.

^h^ Hazardous alcohol use was determined as an AUDIT-C score of ≥4 for males and ≥3 among females.

Abbreviations: N, number; OR, odds ratio; aOR, adjusted odds ratio; CI, confidence intervals; P, p-value; REF, reference; MAT, medication assisted treatment.

### Relationship between baseline HIV drug resistance and viral suppression

As a final step, we evaluated the association between participant characteristics, including HIV drug resistance, and viral suppression (defined as a viral load ≤40 copies/mL after 52 weeks of study follow-up). This analysis included 342 participants who had a baseline resistance result and a viral load result from the week 52 study visit. In both study arms, baseline resistance was associated with a reduced frequency of viral suppression. In the standard of care arm, 2/27 (7.4%) participants with baseline resistance were virally suppressed at week 52, compared to 62/217 (28.6%) participants who did not have resistance (OR: 0.2; 95% CI: 0.05, 0.87; p = 0.032). In the intervention arm, 2/14 (14.3%) participants with baseline resistance were virally suppressed at week 52, compared to 42/84 (50.0%) participants who did not have baseline resistance (OR: 0.17; 95% CI: 0.04, 0.79; p = 0.024). In multivariable analysis, after adjusting for study site and other factors (ARV drugs detected at baseline, baseline viral load, baseline drug resistance, and study arm), three factors were significantly associated with a lower rate of viral suppression: baseline drug resistance, being in the standard of care study arm, and study site (Indonesia vs. Vietnam, Table 4). Viral suppression was significantly lower in Indonesia vs. Ukraine in the univariate model but not in the multivariable model.

**Table 4:** Baseline factors associated with viral suppression.

Variables	Viral Suppression (%)	Unadjusted		Adjusted^a^	
		OR (95% CI)	P	aOR (95% CI)	P
**HIV viral load** (log_10_ copies/mL)^b^		0.95 (0.70, 1.28)	0.740		
**CD4 cell count** (cells/mm^3^)^c^		1.01 (0.91, 1.13)	0.830		
**Baseline HIV drug resistance**					
No	104/301 (34.6%)	REF		REF	
Yes	4/41 (9.8%)	**0.20 (0.07, 0.59)**	**0.003**	**0.16 (0.04, 0.54)**	**0.003**
**Study arm**					
Standard of care	64/244 (26.2%)	REF		REF	
Intervention	44/98 (44.9%)	**2.29 (1.40, 3.74)**	**<0.001**	**2.45 (1.47, 4.08)**	**<0.001**
**Baseline antiretroviral drug(s) detected**					
No	98/303 (32.3%)	REF			
Yes	10/39 (25.6%)	0.72 (0.34, 1.54)	0.398		
**Reported prior/current ART**					
No	74/242 (30.6%)	REF			
Yes	34/100 (34.0%)	1.17 (0.71, 1.92)	0.536		
**Sex**					
Male	91/290 (31.4%)	REF			
Female	17/52 (32.7%)	1.06 (0.57, 1.99)	0.851		
**Age**					
18–29	15/53 (28.3%)	REF			
30–39	68/230 (29.6%)	1.06 (0.55, 2.06)	0.856		
40–60	25/59 (42.4%)	1.86 (0.85, 4.10)	0.123		
**Study site**					
Indonesia	17/88 (19.3%)	REF		REF	
Ukraine	46/133 (34.6%)	**2.21 (1.17, 4.18)**	**0.015**	1.76 (0.90, 3.41)	0.097
Vietnam	45/121 (37.2%)	**2.47 (1.30, 4.71)**	**0.006**	**2.14 (1.09, 4.20)**	**0.026**
**Marital status**					
Married/Have partner but not married	56/174 (32.2%)	REF			
Single/Divorced/Separated/Widowed	52/168 (31.0%)	0.94 (0.60, 1.49)	0.807		
**Education**^d^					
None or primary school	9/27 (33.3%)	REF			
Secondary school	54/171 (31.6%)	0.92 (0.39, 2.19)	0.856		
Higher education	45/144 (31.3%)	0.91 (0.38, 2.18)	0.831		
**Injected amphetamines**^e^ (3 months prior)					
No	91/289 (31.5%)	REF			
Yes	17/52 (32.7%)	1.06 (0.56, 1.99)	0.863		
**Non-injection stimulant use**^e^^,^^f^ (3 months prior)					
No	78/225 (34.7%)	REF			
Yes	29/116 (25.0%)	0.63 (0.38, 1.04)	0.070		
**Non-injection opiate use**^e^^,^^g^ (3 months prior)					
No	98/312 (31.4%)	REF			
Yes	10/29 (34.5%)	1.15 (0.52, 2.56)	0.734		
**Hazardous alcohol use**^h^					
No	65/227 (28.6%)	REF			
Yes	43/115 (37.4%)	1.49 (0.93, 2.39)	0.101		
**MAT for substance use**					
No	30/104 (28.8%)	REF			
Yes	78/238 (32.8%)	1.20 (0.73, 1.99)	0.473		
**Number of sexual partners** (1 month prior)					
0	44/134 (32.8%)	REF			
1	59/185 (31.9%)	0.96 (0.60, 1.54)	0.859		
≥2	5/23 (21.7%)	0.57 (0.20, 1.63)	0.293		
**Number of injection partners** (3 months prior)					
1	16/44 (36.4%)	REF			
2–4	76/256 (29.7%)	0.74 (0.38, 1.44)	0.376		
≥5	16/42 (38.1%)	1.08 (0.45, 2.58)	0.868		
**Jail/Prison (ever)**					
No	104/330 (31.5%)	REF			
Yes	4/12 (33.3%)	1.09 (0.32, 3.69)	0.894		

The table shows factors associated with viral suppression (HIV viral load <40 copies/mL) after 52 weeks of study follow-up. The analysis included 342 index participants who had a baseline HIV drug resistance result and a week 52 viral load result. Logistic regression models were used to estimate adjusted associations between participant characteristics and viral suppression. P-values <0.05 are bolded. For the adjusted models, results are shown for variables that were significantly associated with viral suppression after adjustment for other variables.

^a^ For each variable, univariate associations were adjusted in a multivariable model including covariates: study site, baseline ARV drugs detected (yes/no), baseline viral load, baseline drug resistance (yes/no), and study arm.

^b^ Assessed for increments of log_10_ HIV RNA copies/mL.

^c^ Assessed for increments of 100 CD4-positive cells/mm^3^.

^d^ Secondary school includes completion of some or all of secondary school. Higher education indicates some or complete technical training or college/university education.

^e^ Data for substance use was missing for one participant.

^f^ Stimulants include cocaine and methamphetamines.

^g^ Opiates include heroin and opium.

^h^ Hazardous alcohol use was determined as an AUDIT-C score of ≥4 for males and ≥3 among females.

Abbreviations: N, number; OR, odds ratio; aOR, adjusted odds ratio; CI, confidence intervals; P, p-value; REF, reference; MAT, medication assisted treatment.

## Discussion

We assessed HIV drug resistance and ARV drug use in a large cohort of PWID in Eastern Europe and Asia who were not virally suppressed. In the HPTN 074 trial, index participants in the intervention arm were more likely to be virally suppressed after 52 weeks of follow-up. We identified behavioral and geographic factors associated with baseline resistance in index participants and demonstrated that those with baseline drug resistance were less likely to achieve viral suppression, independent of study arm.

At study entry, 12.0% of the 449 participants had drug-resistant HIV and more than half of the participants with HIV drug resistance had multiclass resistance. The proportion of participants with resistance was significantly higher among those who were using ARV drugs compared to those with no drugs detected (58.8% vs. 6.0%). Participants who reported prior or current ART were more likely to have resistance. However, almost half of those with resistance did not report prior or current ART, even though some of those participants had ARV drugs detected in their study samples. A separate report provides more information on the accuracy of self-reported data in the HPTN 074 cohort [18]. We found an association between resistance and prior incarceration; however, it is important to note that only 14/449 (3.1%) participants reported having been incarcerated. Injection drug use is criminalized in many regions of the world and PWID often face higher incarceration rates; this could impact consistent accessibility to ART and exacerbate development of resistance in this population. This indicates that enhanced HIV care may be particularly important in PWID who have a history of incarceration, and that HIV care programs need to be accessible to those on ART when they are incarcerated, for continuity of care.

The proportion of participants in HPTN 074 who had HIV drug resistance differed by study site (24.1% Indonesia, 2.4% in Ukraine, and 13.4% in Vietnam), which may reflect regional differences in ARV drug use at the time the study was performed. Only 8% of index participants in Ukraine reported prior ART and none had ARV drugs detected at enrollment. In comparison, 56% and 34% of participants in Indonesia and Vietnam reported prior ART and 82% and 53% had ARV drugs detected, respectively. ARV drugs detected were mostly consistent with first-line ART regimens recommended at the study sites at the time the study was performed. Two participants had unusual combinations of ARV drugs detected (two NNRTIs plus an NRTI). Those individuals may have been taking medications without medical guidance. Further research is needed to assess how PWID in these regions access ARV drugs for treatment. We recognize that other local factors, such as recruitment methods or venues, may have influenced the proportion of index participants enrolled who had prior or current ART or other exposures to ARV drugs that could have contributed to resistance.

The prevalence of HIV drug resistance among HPTN 074 participants is likely lower than the prevalence of drug resistance in the general population of PWID in these regions, since HPTN 074 specifically recruited those who were less likely to be on ART. In a previous report that did not use ART history for participant selection, the prevalence of resistance among PWID in Vietnam was much higher (47.4%) [25]. We did not find any prior reports of the prevalence of HIV drug resistance among PWID in Indonesia or Ukraine.

A key feature of this study was that data on HIV drug resistance were analyzed in the context of data from ARV drug testing. This approach allowed us to assess whether the resistance mutations detected were consistent with ARV drugs detected in the same samples. In this cohort, the majority of participants with resistance had mutations detected that confer resistance to the classes of ARV drugs detected. We were also able to assess whether participants were at risk of acquiring additional resistance to additional classes of ARV drugs. In this cohort, 23 participants who were not virally suppressed were taking ARV drugs, but did not have mutations that conferred resistance to one or both classes of ARV drugs detected; acquisition of resistance to those drugs with continued non-suppressive ARV drug exposure would further limit their treatment options.

Data from ARV drug testing and self-report of prior positive HIV status also allowed us to assess whether some of the participants in HPTN 074 may have had transmitted HIV drug resistance (TDR). We detected resistance mutations in samples from 10 (5.6%) of 177 newly-diagnosed participants (6 from Indonesia; 0 from Ukraine; 4 from Vietnam); in all 10 cases, the mutations identified were included in a list of mutations recommended for assessing TDR (hivdb.stanford.edu/s/who). These 10 participants could have had TDR. TDR has been associated with a higher risk of virologic failure among PWID starting ART [26]. In previous reports from Asia, the prevalence of TDR among PWID was 4.0–7.7% (Vietnam: 4.10–7.69% in 2014 [27], 4.0% in 2015 [28]; Indonesia: <5.0% in 2009 [29]). These rates are similar to TDR rates of 2.9%-5.4% reported for PWID in Europe [30–32] and Taiwan [33], and Canada [34] and are lower than the rate of TDR reported among PWID in Iran (~15%) [35]. However, the type of cohort used to assess TDR (e.g., newly-infected individuals, ART-naïve individuals) and the methods used for resistance testing could impact the reported prevalence of TDR.

This study demonstrated that participants in HPTN 074 who had baseline HIV drug resistance were less likely to achieve viral suppression, independent of study arm. The level of viral suppression achieved in both study arms was low (41% in the intervention arm; 24% in the control arm) [14]. Because of the cost and complexity of resistance testing, it is not available in many settings. In HPTN 074, HIV care was provided at local care centers where resistance testing was not routinely performed before initiating ART or at ART failure. This likely contributed to ART failure in some cases.

A limitation of this study is that population (consensus) sequencing is not sensitive for detection of low-level (minority) variants. Another limitation is the possible overestimation of TDR. Our use of an objective measure of ARV drug use in addition to self-report increases the accuracy of TDR estimates. However, the prevalence of TDR may still be overestimated if participants previously used ARV drugs for pre- or post-exposure prophylaxis, prevention of mother-to-child transmission, or other reasons without reporting this to study staff. The ARV drug assay used in this report only detects drugs taken close to the time of sample collection.

In conclusion, this study highlights challenges of using ART for HIV prevention among PWID. Significant resources are needed to identify those with HIV drug resistance, offer appropriate second- or third-line treatment regimens, and provide enhanced adherence support to ensure that these individuals achieve durable viral suppression for their own health and to reduce transmission of drug-resistant HIV to injection and sexual partners. Optimized HIV care is also needed for those starting ART to avoid emergence of resistance strains. A multi-component package for HIV prevention may help prevent HIV transmission from PWID who chose not to start ART or fail ART due to suboptimal adherence, resistance, or other factors. Interventions designed to reduce injection drug use, such as medication-assisted treatment for substance use, may also reduce the risk of superinfection with drug-resistant HIV strains, which could limit treatment options or lead to ART failure. Future studies are planned to assess the impact of baseline resistance on ART outcomes in HPTN 074, and the impact of study interventions (referral and support for ART and medication assisted treatment for substance use) on emergence of resistance during study follow-up.

## Supporting information

S1 Table Detection of HIV drug resistance and antiretroviral drugs. (DOCX)
