# Supplementary material for: HIV drug resistance in persons who inject drugs enrolled in an HIV prevention trial in Indonesia, Ukraine, and Vietnam: HPTN 074
Source: PLoS One. 2019 Oct 10;14(10):e0223829. doi: 10.1371/journal.pone.0223829 (PMC6786608; doi:10.1371/journal.pone.0223829)
Supplement: S1 Table — (DOCX) [file pone.0223829.s001.docx]

**S1 Table. Detection of HIV drug resistance and antiretroviral drugs.**

Group 1: HIV drug resistance and ARV drugs detected (N=30)

| **HIV drug resistance mutations detected**^a^ | | **ARV drugs detected**^b^ | | |
| --- | --- | --- | --- | --- |
| **NNRTI** | **NRTI** | **PI** | **NNRTI** | **NRTI** |
| K103N | - | - | EFV | - |
| K103N | - | - | EFV | - |
| K103N | - | - | EFV | - |
| K103N | - | - | EFV | 3TC* |
| K103N | - | LPV, RTV* | - | 3TC* |
| Y181C | - | - | NVP | - |
| K103N | K65R, M184V | - | EFV | 3TC, TFV |
| K103N | K65R, M184V | - | EFV | 3TC |
| K103N | K65R, M184V | - | EFV | 3TC, TFV |
| K103N | K65R, L74I, M184V | - | EFV | 3TC, TFV |
| K103N | K65R, V75M, M184V | - | EFV | 3TC |
| K103N | V75M, M184V, T215F | - | EFV | 3TC |
| K103N | V75M, M184V, T215Y | - | EFV | 3TC |
| K103N | V75M, M184V, T215Y | - | EFV, NVP | 3TC |
| Y181C | V75M, M184V, T215Y | - | NVP | 3TC, ZDV |
| Y181C | M184V, T215F | - | NVP | 3TC |
| G190A | L74I, M184V, T215Y | - | NVP | 3TC |
| K103N, V106M | M184V, T215F | - | NVP | 3TC |
| K103N, Y181C | M184V | - | EFV | - |
| K103N, Y181C | K65R, M184I | - | EFV | 3TC |
| K103N, G190A | K65R, V75M, M184V | - | EFV | 3TC, TFV |
| K103N, G190S | K65R, V75M, M184V | - | EFV | 3TC |
| K103N, G190S | M184V, T215Y | - | EFV | 3TC, TFV |
| K103N, M230L | M184V | - | EFV | - |
| K103N, M230L | M184V | - | EFV | 3TC |
| V106A, G190A | K65R, V75M, M184V | - | EFV | 3TC, TFV |
| Y181C, G190A | M184V, T215F | - | NVP | 3TC, ZDV |
| Y181C, G190A | M184V, T215F | - | EFV | 3TC |
| Y181C, G190A | K65R, V75M, M184V | - | NVP | 3TC |
| K101P, K103N, G190A | M184V | - | EFV | 3TC |

Group 2: ARV drugs detected with no HIV drug resistance detected^b^ (N=21)

| **HIV drug resistance mutations detected** | | **ARV drugs detected** | | |
| --- | --- | --- | --- | --- |
| **NNRTI** | **NRTI** | **PI** | **NNRTI** | **NRTI** |
| - | - | - | EFV | - |
| - | - | - | NVP | - |
| - | - | - | NVP | - |
| - | - | - | NVP | - |
| - | - | - | EFV | 3TC |
| - | - | - | EFV | 3TC |
| - | - | - | EFV | 3TC |
| - | - | - | EFV | 3TC |
| - | - | - | EFV | 3TC |
| - | - | - | EFV | 3TC |
| - | - | - | EFV | 3TC |
| - | - | - | EFV | 3TC |
| - | - | - | EFV | FTC |
| - | - | - | EFV | 3TC, TFV |
| - | - | - | EFV | 3TC, TFV |
| - | - | - | EFV | 3TC, TFV |
| - | - | - | EFV | 3TC, TFV |
| - | - | - | EFV | 3TC, ZDV |
| - | - | - | EFV | 3TC, ABC |
| - | - | - | EFV, NVP | 3TC |
| - | - | LPV, RTV | - | TFV, FTC |

Group 3: HIV drug resistance with no ARV drugs detected (N=24)

| **HIV drug resistance mutations detected** | | **ARV drugs detected** | | |
| --- | --- | --- | --- | --- |
| **NNRTI** | **NRTI** | **PI** | **NNRTI** | **NRTI** |
| K103N | - | - | - | - |
| K103N | - | - | - | - |
| K103N | - | - | - | - |
| K103N | - | - | - | - |
| K103N | - | - | - | - |
| K103N | - | - | - | - |
| K103N | - | - | - | - |
| K103N | - | - | - | - |
| K103N | - | - | - | - |
| K103N | - | - | - | - |
| K103N | - | - | - | - |
| K103N | - | - | - | - |
| K103N | - | - | - | - |
| K103S | - | - | - | - |
| Y181C | - | - | - | - |
| Y181C | - | - | - | - |
| G190S | - | - | - | - |
| K103N, Y181C | - | - | - | - |
| K103N, Y181C | - | - | - | - |
| K103N | M184V | - | - | - |
| K103N | M184V | - | - | - |
| K103N | L74I, M184V, T215Y | - | - | - |
| K101P, K103N | K65R, V75M, M184V | - | - | - |
| G190S | M184V | - | - | - |

Footnotes for S1 Table.

^a^ Major resistance mutations are shown.

^b^ In some cases, antiretroviral (ARV) drugs were detected in samples that did not have major resistance mutations corresponding to the classes of those ARV drugs. This includes two participants in Group 1 (noted with an asterisk) and the 21 participants in Group 2. These 23 participants were at risk of acquiring resistance to the corresponding classes of ARV drugs.

Abbreviations: ARV, antiretroviral; N, number; NNRTI, non-nucleoside reverse transcriptase inhibitor; NRTI, nucleoside/nucleotide reverse transcriptase inhibitor; PI, protease inhibitor; EFV, efavirenz; 3TC, lamivudine; LPV, lopinavir; RTV, ritonavir; NVP, nevirapine; TFV, tenofovir; ZDV, zidovudine; FTC, emtricitabine; ABC, abacavir.
